# The Importance of Lipoprotein Lipase Regulation in Atherosclerosis

**DOI:** 10.3390/biomedicines9070782

**Published:** 2021-07-06

**Authors:** Anni Kumari, Kristian K. Kristensen, Michael Ploug, Anne-Marie Lund Winther

**Affiliations:** 1 Finsen Laboratory, Rigshospitalet, DK-2200 Copenhagen N, Denmark; Anni.Kumari@finsenlab.dk (A.K.); kristian.kristensen@finsenlab.dk (K.K.K.); m-ploug@finsenlab.dk (M.P.); 2 Biotech Research and Innovation Centre (BRIC), University of Copenhagen, DK-2200 Copenhagen N, Denmark

**Keywords:** lipoprotein lipase, ANGPTL, atherosclerosis

## Abstract

Lipoprotein lipase (LPL) plays a major role in the lipid homeostasis mainly by mediating the intravascular lipolysis of triglyceride rich lipoproteins. Impaired LPL activity leads to the accumulation of chylomicrons and very low-density lipoproteins (VLDL) in plasma, resulting in hypertriglyceridemia. While low-density lipoprotein cholesterol (LDL-C) is recognized as a primary risk factor for atherosclerosis, hypertriglyceridemia has been shown to be an independent risk factor for cardiovascular disease (CVD) and a residual risk factor in atherosclerosis development. In this review, we focus on the lipolysis machinery and discuss the potential role of triglycerides, remnant particles, and lipolysis mediators in the onset and progression of atherosclerotic cardiovascular disease (ASCVD). This review details a number of important factors involved in the maturation and transportation of LPL to the capillaries, where the triglycerides are hydrolyzed, generating remnant lipoproteins. Moreover, LPL and other factors involved in intravascular lipolysis are also reported to impact the clearance of remnant lipoproteins from plasma and promote lipoprotein retention in capillaries. Apolipoproteins (Apo) and angiopoietin-like proteins (ANGPTLs) play a crucial role in regulating LPL activity and recent insights into LPL regulation may elucidate new pharmacological means to address the challenge of hypertriglyceridemia in atherosclerosis development.

## 1. Introduction

There are three main lipids in the blood: cholesterol, phospholipids, and triglycerides. Cholesterol is important for the synthesis of bile acids and steroids and for maintaining the integrity of cell membranes, while phospholipids are a major component of all cell membranes. Triglycerides (TGs) serve as a storable high-energy fuel. In humans, almost 80% of the energy requirements of the heart and liver are fulfilled by oxidation of the long fatty acyl chains present in TGs [1].

**Figure 1:**
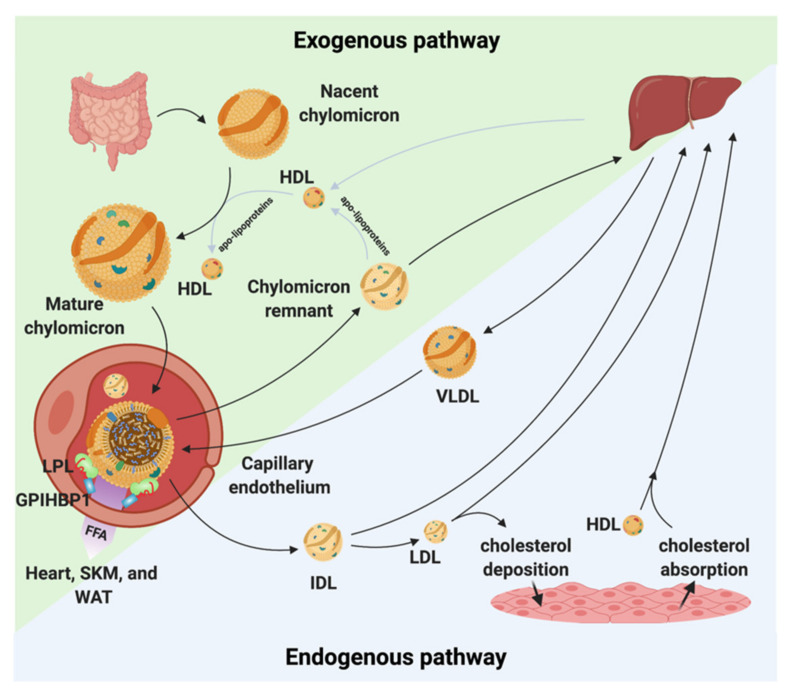
Chylomicron and VLDL life cycle. Chylomicrons are secreted from the intestines to the circulation where they extract apolipoproteins from HDL. The mature chylomicrons are arrested at the capillary endothelium by LPL that is in complex with glycosylphosphatidylinositol anchored high density lipoprotein binding protein 1 (GPIHBP1). LPL performs lipolysis on the encapsulated TGs, which releases free fatty acids that are taken up by the underlying tissues (white adipose tissue (WAT), skeletal muscle (SKM), and the heart). Upon complete hydrolysis, a chylomicron remnant particle is released to the circulation. The chylomicron remnant particle back-exchanges apolipoproteins to circulating HDL, before final catabolism in the liver. A similar life cycle is found for VLDL. VLDL is secreted from the liver and is arrested and hydrolyzed by the LPL•GPIHBP1 complex. This releases IDL, which can either be taken up by the liver or be hydrolyzed further into LDL. LDL deposits cholesterol esters in various tissues. HDL absorbs cholesterol and transports it to the liver.

Because lipids are poorly soluble in blood plasma, they need to be transported as lipoproteins. Lipoproteins are composed of cholesteryl esters and TGs surrounded by a hydrophilic shell made of phospholipids, unesterified cholesterol, and apolipoproteins. Depending upon the origin of the lipoproteins they differ in size, density, and composition [2,3]. The lipoproteins are generally divided into five major classes. These classes are chylomicrons, VLDL, LDL, intermediate-density lipoprotein (IDL), and high-density lipoproteins (HDL). Each class of lipoprotein serves distinct roles in the lipid metabolism (Figure 1); *(i)* the large chylomicrons and VLDLs are responsible for the transport and delivery of energy rich TGs, *(ii)* LDL deposits cholesterol in tissues, and *(iii)* HDL absorbs cholesterol and transports it back to the liver for degradation and redistribution (Figure 1). The lipoproteins constantly undergo enzymatic processing, exchange of lipids or apolipoproteins, and progressive degradation by lipid unloading. Combined, this network is crucial for lipid homeostasis, and even minor alternations in either of the lipoproteins classes potentially perturbs the entire lipoprotein processing network. LDL and HDL have been found to be important for the development of arteriosclerosis due to their cholesterol cargo. However, studies have shown that elevated plasma triglyceride levels known as hypertriglyceridemia (HTG) is associated with an increased risk of atherosclerosis and CVD [4,5,6]. LPL is responsible for the hydrolysis of the triglyceride in capillaries and thereby transform chylomicrons and VLDL to smaller lipoproteins particles. This process is tightly regulated by a number of proteins that either display a stabilizing, activating, or inhibitory effect of LPL and perturbations of this homeostasis may have anti-atherogenic effects [7,8,9,10,11]. On the other hand, data have shown that when LPL is expressed by macrophages in the vessel wall it displays pro-atherogenic effects [12]. With this in mind, there is a great need to understand the underlining molecular mechanism(s) causing increased triglyceride levels in hypertriglyceridemia and atherosclerosis and map the complex regulation of LPL. This knowledge will likely identify new avenues for lipid lowering strategies that could serve as supplements to the existing interventions (e.g., statins) to help further restraining ASCVD progression.

## 2. The Lipoprotein Lipase

**Figure 2:**
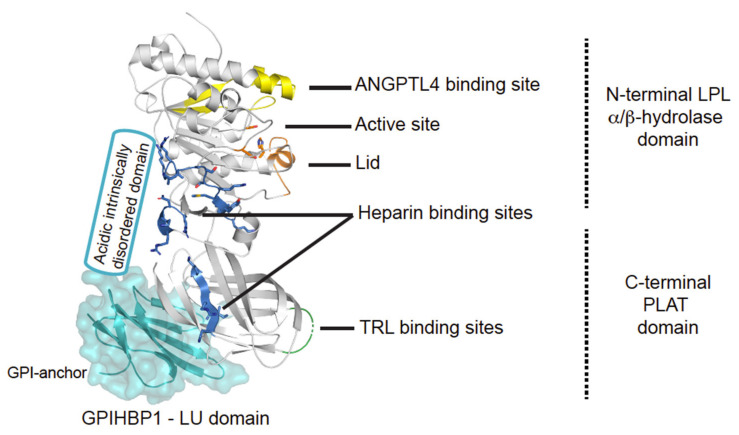
Structural elements in LPL. Cartoon representation of the human LPL•GPIHBP1 complex and with the molecular surface of the LU domain (Ly6/uPAR protein domain family) of GPIHBP1 shown as a transparent light blue envelope (generated with The PyMol Molecular Graphic System (Version 2.0 Schrödinger, LLC) using coordinates from the LPL•GPIHBP1 crystal structure; Protein Data Bank ID code 6E7K). LPL elements implicated in ANGPTL4 binding are highlighted in yellow [17]. GPIHBP1 binds to the C-terminal PLAT domain of LPL, and the location of GPIHBP1’s membrane-tethering site (GPI anchor) is indicated [18]. GPIHBP1’s acidic intrinsically disordered domain stabilizes LPL’s α/β-hydrolase domain and is assumed to interact with a large basic patch on the surface of LPL [19,20]. The active site containing the catalytic triad (Ser134, Asp158, and His243) is represented with orange residues [21] and the orange helix is the lid covering the active site responsible for substrate specificity [22]. The triglyceride-rich lipoprotein (TRL) binding site is indicated in green and residues important for heparin binding is shown in blue [23,24,25].

The central role of LPL in triglyceride hydrolysis was recognized over 60 years ago by the discovery of the enzyme in 1955 [13]. The expression level of LPL is robust in cells and organs with a high oxidative metabolism, but it is also expressed in other tissue types, not related to intravascular lipolysis such as the spleen, testis, lung, kidneys, and brain as well as in macrophages [14]. LPL is a 55 kDa glycoprotein consisting of an N-terminal α/β-hydrolase domain and a C-terminal Polycystin-1, Lipoxygenase, Alpha-Toxin (PLAT) domain (Figure 2). The α/β-hydrolase domain harbors the active site that primarily hydrolyses the sn-1/sn-3 ester bonds of triglycerides, thereby releasing two unesterified fatty acids and a sn-2 monoacylglycerol [15]. The hydrolysis takes place at the luminal face of the capillary endothelium where LPL hydrolyzes the triglycerides within the neutral cores of chylomicrons and VLDLs producing chylomicron remnants and IDLs, respectively (Figure 1) [16]. The released free fatty acids (FFA) can either be used as an energy source or be re-esterified and stored in adipose tissues as triglycerides.

### 2.1. Maturation and Transportation of LPL

**Figure 3:**
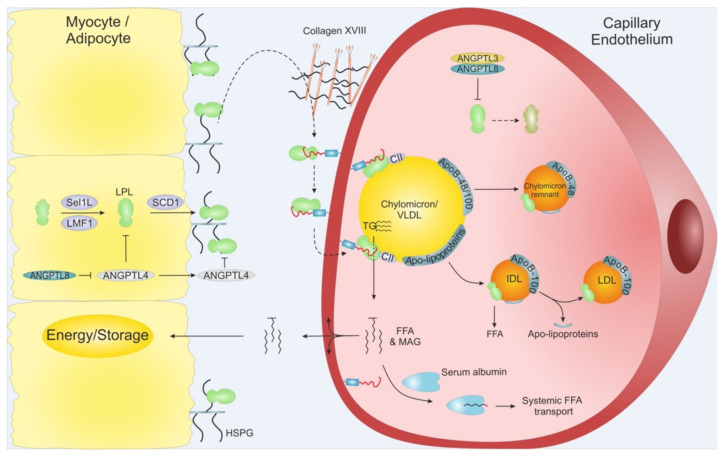
Schematic overview of LPL translocation. LPL is secreted from adipose tissue and myocytes from skeletal muscles and heart. Intracellular LPL folding and secretion is assisted by LMF1, Sel1L and SCD1. After secretion, active LPL is bound to cell surface HSPG before being translocated to GPIHBP1, potentially via Collagen XVIII. GPIHBP1 captures LPL in the subendothelial space and mediates LPL trancytosis across the capillary endothelium, into the lumen. Within the lumen, the LPL•GPIHBP1 complex is responsible for the arrest of VLDLs and chylomicrons. After final hydrolysis, either a chylomicron remnant particle or an IDL are released to be taken up by the liver or turned into LDL via further TG hydrolysis, respectively. ANGPTL4 act as a potent LPL inhibitor in the adipose tissue, and ANGPTL3•ANGPTL8 act on LPL mainly in the capillaries of the muscles and heart. The inhibition of the ANGPTLs is driven by nutritional status and exercise. MAG: Monoacylglycerol.

Adipocytes and myocytes are the prime sources of LPL production and because these cells are located distantly from the capillary lumen, where LPL acts on marginated lipoproteins, it needs to be trafficked across the subendothelial space and trancytosed across the endothelial cells [14,26]. Accordingly, LPL secretion requires several chaperones and transporter proteins to ensure correct shuttling and folding of LPL [27,28,29,30]. One such protein is the lipase maturation factor (LMF1), which is essential for LPL processing since loss-of-function variants in *LMF1* cause hypertriglyceridemia due to inefficient LPL secretion [28,31]. Besides LMF1, the suppressor of lin-12-like protein 1 (Sel1L) and syndecan-1 (SCD1) have been shown to be vital for LPL secretion (Figure 3) [29,30].

Upon successful secretion, LPL becomes tethered to cell surface heparans sulfate proteoglycans (HSPGs) via its heparin binding sites (Figure 2 and Figure 3) [23,24,25]. HSPG-bound LPL provides a storage pool that can be rapidly translocated to the capillary lumen via the endothelial cell surface bound protein GPIHBP1 (Figure 3). The movement of LPL towards GPIHBP1 is currently controversial and has been suggested to include random diffusion [32], directed diffusion on HSPG sulfation-gradients [19], and heparanase mediated release [33]. Interestingly, collagen XVIII with its heparin sulfate modifications may act as the proximal reservoir of LPL on the abluminal face of the capillary endothelium since *Col18a1*^−/−^ mice have increased plasma TGs levels and decreased plasma LPL mass [34].

Once bound to GPIHBP1, LPL can move bidirectionally between the luminal and abluminal surfaces of the endothelial cells by caveolin1-independent transcytosis [35,36]. The expression of GPIHBP1 is restricted to capillary endothelial cells—it is absent in larger vessels—rendering the capillaries the sole relevant site for intravascular lipolysis [26,37]. GPIHBP1 contains a folded LU domain with ten consensus cysteines and a disordered acidic domain (Figure 2) [20,38]. The pivotal role of GPIHBP1 in intravascular lipolysis is emphasized by the fact that a fraction of patients suffering from chylomicronemia are homozygous or combined biallelic heterozygous for *GPIHBP1* missense variants that encode a non-functional GPIHBP1 protein [39,40,41,42,43]. In these patients, the binding between GPIHBP1 and LPL is impaired, and hence LPL activity is absent in the lumen of the capillary. Further underscoring the importance of GPIHBP1, is a subset of patients with hitherto unexplained, late-onset acquired chylomicronemia that had a new disease etiology—auto-immunity against GPIHBP1 with auto-antibodies preventing LPL-translocation into capillary lumen [44]. Recently, it has been shown that this disease severity can be ameliorated in some patients with immunosuppressive therapy [45].

### 2.2. Enzymatic Function of LPL

Once translocated to the luminal face of the capillary endothelium, the LPL•GPIHBP1 complex is responsible for the margination (arrest) of the circulating triglyceride-rich lipoprotein (TRL) particles [46]. In *Gpihbp1*^−/−^ mice, the TRLs do not marginate if LPL is absent from the luminal endothelium. The systemic administration of bovine LPL intravenously or the transgenic expression of human LPL in endothelial cells do not induce TRL magination in *Gpihbp1*^−/−^ mice [46]. The binding to TRLs is exclusively mediated by LPL, not GPIHBP1 (Figure 2) [46,47]. Although the molecular mechanism(s) of LPL-mediated TRL processing is not completely understood, several regions of the lipase molecule are known to be essential for its activation, TRL binding and substrate specificity [18,48,49,50,51,52,53].

The LPL-mediated hydrolysis of TGs in the core of TRLs is extremely efficient. In vivo imaging studies with ^2^H-triglycerides showed that margination, TG hydrolysis, and the subsequent uptake of free fatty acids occur within a few minutes in perfused hearts [54]. These observations are in line with kinetic data from in-vitro studies on TRL processing in solution, which estimate that on average 44 LPL molecules engage simultaneously in the lipolytic processing each TRL particle [15]. Electron microscopy of cardiomyocytes show that GPIHBP1•LPL complexes are located on small membrane protrusions (“nanovilli”) lining the TRL particle. This anatomical structure allows for a larger contact area with the TRLs and enables the concomitant engagement of a larger number of LPL molecules in the lipolytic processing [46].

It is currently debated exactly how the released FFA are taken up by the underlying tissues. Observing ^2^H-free fatty acids in the cytoplasma of cardiomyocytes a few minutes after margination indicates that they rapidly diffuses across the plasma membrane into the underlying cells [54]. It was suggested previously that CD36 actively transports FFAs. However, the genetic ablation of CD36 did not change the fast kinetics of ^2^H-FFA uptake in cardiomyocytes [54]. In addition to the local FFA uptake, binding to serum albumin allows the FFA to be transported and used at sites distant from the site of hydrolysis [15]. If the generated FFAs are not removed locally they will auto-inhibit LPL and slow down the lipolysis [15]. Release of high amount of FFAs by systemic lipolysis may cause inflammation and lipotoxicity [55].

### 2.3. LPL Is Active as a Monomer

Despite intensive research efforts, the structural insights into LPL function remained elusive for decades primarily due to the low stability of the enzyme. Perceptions of the structure–function relationships in LPL relied largely on homology modeling using the crystal structure of pancreatic lipase (PL) as a template [50]. Although these homology considerations provided some useful information, the low sequence identity between PL and LPL (27%) paired with PL’s need for a cofactor (co-lipase) limits the confidence of such homology models [56]. From a structural perspective, the discovery of GPIHBP1 and the realization that GPIHBP1 stabilizes LPL was decisive for solving the crystal structure of LPL [20,36,57]. In short succession, two independent groups published virtually identical X-ray structures of LPL in complex with GPIHBP1 [18,58]. In both structures, LPL formed the expected head-to-tail homodimer. That dimeric LPL configuration was widely accepted to represent the active conformation of LPL, and the individual monomers thought to be unstable and inactive [59,60]. In the crystal structure, the reciprocal dimer interface was, however, formed by the burial of the TRL substrate binding tryptophans of one protomer in the active site cleft of the partner protomer [18,58]. That observation raised the question: How does the dimeric LPL allows substrate entry when the active site is blocked by the TRL binding loop? This conundrum was, however, quickly resolved as LPL monomers rather than LPL dimers were found to represent the active LPL conformation [61]. This shift in the paradigm on the active LPL conformation was subsequently supported by biophysical measurements showing that LPL monomers and LPL dimers had equal stability, esterase activity and inhibitory profile when exposed to ANGPTL4 [62].

### 2.4. GPIHBP1-Binding Render LPL Stable at Body Temperature

In recent years, it has become increasingly clear that LPL’s inherent protein instability plays a key role for the regulation of intravascular lipolysis. Early studies reported the progressive loss of LPL activity at 37 °C, and that the presence of heparin preserved enzymatic activity [60,63]. Studies with hydrogen-deuterium exchange coupled to mass spectrometry (HDX-MS) provided the first details on the molecular mechanism(s) underlying this instability. The Achille’s heel of the LPL protein weakening its thermal stability is found in the α/β-hydrolase domain, which unfolds spontaneously [17,20,53,62]. In contrast, the C-terminal PLAT domain is stable. The global unfolding observed with HDX-MS was confirmed by differential scanning fluorimetry (DSF) showing that the catalytic α/β-hydrolase domain unfolds with a *T_m_* of 34.8 °C, which is well below normal body temperature [17]. Binding of either GPIHBP1 or HSPGs stabilize the α/β-hydrolase domain profoundly (T_m_ = 57.6 °C or 42.2 °C, respectively). Importantly, ANGTPL4 has the opposite effect on LPL stability decreasing T_m_ for unfolding of the α/β-hydrolase domain to less than 15 °C for LPL alone and to 36.6 °C for LPL•GPIHBP1 complexes. Another event stabilizing LPL is the binding to lipoproteins, which also mitigates ANGPTL4 and ANGPTL3 mediated inhibition [64]. In aggregate, these studies show that LPL auto-inactivates by spontaneous unfolding, unless its intrinsic instability is counteracted by binding to GPIHBP1 or HSPG. This weakness is further exploited by ANGPTL3 and ANGPTL4 that inhibit LPL by catalyzing the unfolding of its catalytic domain [65,66].

In normal subjects, plasma LPL mass does not correlate with LPL activity since most plasma LPL is inactive. Upon heparin administration, active LPL is efficiently released causing mass and activity to correlate [67,68]. As mentioned, inactive LPL was widely accepted to represent LPL monomers, but now we understand that the inactive form most likely represents partly unfolded LPL monomers, with a folded PLAT domain [20,61]. This scenario has implications such as, *(i)* antibodies recognizing that active LPL is unlikely to bind the unfolded catalytic domain of inactive species in plasma and *(ii)* interactions with inactive LPL in circulation would be expected to involve the relatively thermostable PLAT domain. In fact, this is in line with evidence suggesting that the PLAT domain of inactive LPL is responsible for remnant uptake via LDL receptor related protein 1 (LRP1) and LDL-receptor (LDL-R) [69,70,71]. Lipoprotein profiling suggests that both inactive and active LPL associates mainly with LDL and HDL in both pre- and post-heparin plasma, whereas active LPL associates predominantly with TRLs when it is released with heparin in the post-prandial state [67]. Furthermore it has been suggested that inactive LPL preferably binds native LDL, while active LPL preferentially binds oxidized LDL [72]. How these differences in binding preferences between active and inactive LPL relate to the conformational stability of LPL’s α/β hydrolase domain is currently unexplored.

## 3. LPL’s Activity Is Regulated by Apolipoproteins

Apolipoproteins are integrated in the maintenance of lipid homeostasis. They constitute a class of amphipathic proteins having a hydrophobic region that interacts with lipids in the lipoprotein particles, while the hydrophilic region allows interaction with soluble proteins. They provide structure-defining elements for lipoprotein particles and serve as ligands for specific receptors. Both ApoC-II and ApoA-V act as positive regulators of LPL in intravascular lipolysis, while ApoC-I and ApoC-III display an inhibitory role towards LPL. Apolipoproteins may promote atherogenic or anti-atherogenic effects depending on their expression levels and microenvironment.

### 3.1. Apolipoprotein C-II Enhances LPL Activity

ApoC-II is the only known and essential cofactor for LPL in mediating the hydrolysis of TRLs [73,74]. In plasma, ApoC-II is found associated to VLDL, chylomicrons and HDL particles [75,76]. It contains 79 amino acids arranged in three α-helices and the third C-terminal helix interacts with LPL [77,78,79]. Chemical cross-linking and site directed mutagenesis studies have provided some insights into how ApoC-II interacts with LPL. Four specific amino acid residues (Tyr63, Ile66, Asp69, and Gln70) in the C-terminal helix of ApoC-II have been shown to be important for interaction with LPL [80], but the amino acid residues of LPL that are supposed to interact with ApoC-II have not been fully resolved yet [81,82]. Recently, it was proposed that ApoC-II regulates LPL activity in a pressure dependent model [83]. In this model, ApoC-II remains attached to the VLDL and chylomicron surfaces during LPL mediated TG hydrolysis. As LPL consumes the neutral core TGs, the surface pressure on TRLs increases. Once this surface pressure is greater than a given ApoC-II retention pressure, it leads to the extrusion of ApoC-II from the TRL. An unresolved paradox in Apo-CII biology is that over-expression of ApoC-II leads to HTG, due to decreased lipolysis [84]. Using the pressure dependent model, we can therefore hypothesize that high levels of ApoC-II on the TRL surface would increase the surface pressure preventing LPL binding, thereby attenuating lipolysis, when ApoC-II is overexpressed.

One of the pathogenic properties of ApoC-II is its ability to form amyloid fibrils by undergoing self-association in the absence of lipids [85]. In vivo studies have demonstrated the accumulation of these fibrils in atherosclerotic plaques in the arteries [86]. These amyloid fibrils were reported to initiate CD-36 dependent signaling resulting in macrophage-mediated inflammatory response such as production of tumor necrosis factor-α and reactive oxygen species (ROS)—a crucial event in the progression of atherosclerosis [86]. Accordingly, ApoC-II was shown to co-localize with macrophages in murine arterial lesions [87]. Due to its pivotal role in maintaining lipid homeostasis, ApoC-II has attracted considerable attention as a potential therapeutic strategy in CVD [88]. Although the mechanistic insights of ApoC-II and its association with atherosclerosis are not yet fully elucidated, further investigations in this field may lead to new therapeutic potentials aimed at ASCVD.

### 3.2. Apolipoprotein A-V as Positive Regulator of LPL Mediated Lipolysis

ApoA-V was first identified by Vliet and colleagues [89]. In vivo studies showed that ApoA-V^−/−^ mice had four times higher serum TG levels compared to littermate controls, indicating a possible role in TG metabolism [90]. An independent study on ApoA-V^−/−^ mice confirmed the beneficial impact of ApoA-V on TG hydrolysis and clearance of lipoprotein remnants [91]. This impact was underscored by lowered TG levels in transgenic mice overexpressing ApoA-V [92]. In humans, genome-wide association studies (GWAS) found associations between common variants of *APOA5* and HTG [93]. In aggregate, ApoA-V has a positive impact on intravascular lipid homeostasis.

ApoA-V is only expressed in the liver [89] and is found integrated in circulating VLDL, chylomicrons, and HDL particles [94]. It is highly hydrophobic and its mature form (343 amino acids) is predicted to have a high α-helix propensity [95]. With a view to its pronounced impact on intravascular lipolysis homeostasis, the plasma concentration of ApoA-V is intriguingly low (24–400 ng/mL), which equals one ApoA-V molecule per 1000 VLDL [94].

Circumstantial biochemical evidence suggests that ApoA-V may interact with LDL-R family receptors and GPIHBP1, but the functional impact of such interactions remains unclear primarily due to the vast molar excess of these potential binding partners [36,96]. In vitro assays suggest that ApoA-V at concentration >3 µM inhibits LPL, which is supraphysiologic concentration and in vivo effect is therefore questionable [97]. A more plausible mode of action for ApoA-V in intravascular lipolysis has recently been formulated by Chen and coworkers, who took the low concentrations of plasma ApoA-V into account in their model [98]. They reported that ApoA-V interacts specifically and with very high affinity with ANGPTL3/ANGPTL8 complexes and in doing so it abrogates the LPL inhibitory effect of that complex [98].

The impact of ApoA-V on TG metabolism and atherosclerosis is also evident from in vivo mice studies [99,100]. In a hypercholesterolemic mouse model (*ApoE*^−/−^), the presence of ApoA-V reduced both TG and cholesterol levels in plasma when they were fed a regular chow diet and the clearance rate of remnant particles was increased two-fold higher [100,101]. HSPGs, most notably SCD1, are prominent endocytic receptors in liver involved in the clearance of TRL remnants [102,103]. Recently, it has been reported that ApoA-V binds to HSPGs and mediates the clearance of TRL remnant particles [101] and thus likely reducing the risk of atherosclerosis.

Given its important role in lipid metabolism, ApoA-V holds a strong therapeutic potential. In that setting, increasing plasma levels of ApoA-V by an intravenous injection of recombinant ApoA-V or by adeno-associated virus mediated gene transfer was beneficial in treating HTG [104,105,106].

### 3.3. Overexpression of Apolipoprotein C-I Leads to Severe HTG in Both LPL Dependent and LPL-Independent Manner

ApoC-I is the smallest and most basic of all the apolipoproteins [107] containing 57 amino acids in its mature form [108,109]. In plasma, ApoC-I is found associated to HDL during fasting, while in the postprandial state it moves from HDL to VLDLs and chylomicrons [110,111]. Despite being a small protein with two short α-helices, ApoC-I has a pleiotropic effect on lipoprotein metabolism [112]. It inhibits multiple enzymes involved in lipid metabolism such as LPL, hepatic lipase, phospholipase A2, and cholesteryl ester transfer protein (CETP) [113,114,115,116]. It also mediates the efflux of cholesterol from macrophages [117]. In vivo studies suggest that LPL inhibition by ApoC-I causes TG levels to increase in the setting of ApoC-I overexpression [113,118]. Another mechanism by which ApoC-I increases TG levels is by inhibiting ApoE-mediated hepatic uptake of IDL and VLDL via LRP1 and LDL-R [119,120]. ApoC-I also regulates HDL levels as it inhibits HDL uptake by scavenger receptor class B type 1 (SRB1) [121]. Finally, ApoC-I is a relatively poor activator of lecithin-cholesterol acyltransferase (LCAT), which is the enzyme responsible for converting cholesterol to cholesteryl ester [122]. Due to this pleiotropic effect on TGs and cholesterol regulation, investigators have tried to dissect ApoC-I’s potential role in atherosclerosis, which appears to be highly context dependent. A number of observations indicate that ApoC-I levels are elevated in atherosclerotic lesions which is predictive for disease progression and has a bearing on foam cell formation [87,123]. Increased ApoC-I content in remnant particles has been proposed as an independent marker for estimating risk of coronary heart disease (CHD) [124,125]. In postprandial TRLs, increased ApoC-I level is also associated with increased cholesterol content, further bolstering its role in atherosclerosis [125]. Another study reports reduction in LDL uptake by macrophages upon silencing of ApoC-I mRNA strengthening the pro-atherosclerotic role of ApoC-I [126]. Paradoxically, systemic ApoC-I levels are positively correlated to risk of atherosclerosis [117], while decreased level of ApoC-I in HDL particles is observed in patients with CHD [127].

Transgenic rabbits expressing human ApoC-I displayed a 22% reduction in atherosclerotic lesions and increased plasma HDL cholesterol when fed a diet high in cholesterol [128]. Rabbits on chow diet also displayed increased levels of HDL cholesterol (HDL-C) and 14% decrease in specific CETP activity [128]. Due to these deviating results, it is difficult to unambiguously assign the impact of ApoC-I as pro- or anti-atherosclerotic.

### 3.4. Apolipoprotein C-III Inhibits LPL and Blocks Hepatic Uptake of Remnant Particles

ApoC-III is one of the key regulators inhibiting triglyceride metabolism. Studies combining human genetics and epidemiology reveal that heterozygous missense variants in *APOC3* are associated with a 44% decrease in non-fasting TGs levels and a significant reduction in the risk of CHD and ischemic vascular disease incidents [129]. Further consolidating this association, life-long deficiency of ApoC-III has a cardioprotective and anti-atherosclerotic profile [130], while increased ApoC-III level is associated with a higher risk of CHD [131]. ApoC-III contains 79 amino acids and associates mainly to VLDL, chylomicrons, HDL particles, and to a lower degree with LDL [132]. It inhibits LPL activity [133,134,135] and blocks the hepatic uptake of remnant particles [136]. Accordingly, elevated levels of ApoC-III in mice and humans are correlated to increased TG levels [131,137]. ApoC-III assists in the intracellular synthesis, assembly, and secretion of VLDLs [138]. In hypertriglyceridemia caused by elevated ApoC-III levels, the VLDL concentration in plasma increases due to a dual impact of ApoC-III: *(i)* decreased LPL activity and *(ii)* enhanced synthesis and hepatic secretion of VLDL. Although the molecular mechanism of LPL inhibition by ApoC-III are not fully elucidated, it may involve the competitive displacement of other apolipoproteins from the surface on TRL particles [139]. The involvement of ApoC-III in key steps of lipoprotein metabolism makes it a potential target for therapeutics aimed at treating residual CVD and atherosclerosis. Volanesorsen, is one such second-generation drug, targeting ApoC-III expression with antisense probes. As a supplement to dietary restrictions, it has proven highly beneficial in patients with familial chylomicronemia syndrome (FCS) lowering TGs levels by 70–80% [140,141] and this represents a promising start for future drugs targeting ApoC-III.

## 4. Regulation of LPL Lipolytic Activity by ANGPTLs

Intravascular lipolysis is strictly regulated by specific moderators of LPL activity. A tight temporal and spatial regulation of LPL ensures that the flux of FFA arrives at the right time in target tissues in need of energy supply. This anatomical regulation is mediated by members of the angiopoietin-like (ANGPTL) family, ANGPTL3, ANGPTL4, and ANGPTL8 (Figure 3) [142]. ANGPTL3 and ANGPTL4 are both composed by an N-terminal coiled-coil domain (CCD), a linker region, and a C-terminal fibrinogen-like domain (FLD), while ANGPTL8 lacks a FLD. ANGPTL3 and ANGPLT4 both inhibit LPL, but with vastly different efficacy, and recent studies have expanded the complexity of LPL regulation as ANGPTL8 binding increased the potency of ANGPTL3 and reduces that of ANGPLT4. Like other key players in TG metabolism, the atherosclerotic profiles for ANGPTLs are context dependent, highlighting the importance of understanding their biological and biochemistry.

### 4.1. ANGPTL4 Inhibits LPL Activity in WAT in the Fasting State

Human ANGPTL4 is a 50 kDa glycoprotein highly expressed in WAT, but also in other tissues/cell types such as liver and in macrophages [143]. The expression of ANGPTL4 is induced by exercise or low serum lipid levels [144,145,146,147]. This ensures that LPL activity in WAT is suppressed when serum TGs levels are low (fasting), prioritizing energy uptake in oxidative tissues. Conversely, in the fed state, where serum lipid levels are abundant, ANGPTL4 expression is decreased in WAT, allowing replenishing of the energy storage [147,148]. ANGPTL4 levels directly affect plasma TGs levels as shown in *Angptl4*^−/−^ mice that have low serum TG levels as a consequence of unchecked LPL activity in WAT [147,148]. The observation that levels of circulating ANGPTL4 rarely correlate with plasma TG levels appeared at first sight enigmatic [10,149,150]. This paradox was, however, rooted in the intracellular action of ANGPTL4 in WAT where it primes LPL to degradation by promoting PCSK3 cleavage and subsequent degradation [146,151,152]. Recently, it was suggested that ANGPTL4-mediated unfolding of LPL sensitized it to cleavage by PCSK3 [65]. Notwithstanding this intracellular effect, freshly secreted LPL bound to cell surfaces in the subendothelial space is also targeted by ANGPTL4 [57,153].

The molecular mechanism of ANGPTL4-mediated LPL inhibition represents a novel mode of action for enzyme inhibition. ANGPTL4 binds close to the active site in LPL and this binding event triggers the progressive and irreversible unfolding of LPL’s α/β hydrolase domain [17,59,62,154]. Accordingly, ANGPTL4 was dubbed a “molecular unfolding chaperone” and its catalytic function is evident by the fact that sub-stoichiometric amounts of ANGPTL4 over time causes a complete LPL inhibition in vitro [59,154]. Importantly, this irreversible inactivation by unfolding is mitigated by binding to GPIHBP1 [17,57,154,155].

In human genetic and epidemiologic studies, ANGPTL4 was shown to affect plasma TGs levels. Function impairing missense variations in *ANGPTL4* (e.g., *ANGPTL4 E40K)* thus correlated with increased plasma levels of TGs and HDL-C and furthermore decreased the risk of CVD [9,10,156,157]. As the corresponding ANGPTL4-E15K protein variant has impaired inhibition of LPL [154,157], these studies advance the proposition that a systemic lowering of the plasma TGs, by boosting LPL activity via a reduced ANGPTL4 inhibition, has therapeutic potential. Unfortunately, *Angptl4*^−/−^ mice kept on a high fat diet becomes moribund due to excessive intestinal inflammation and fibrosis [158]. This unexpected pathology arose, since ANGPTL4 prevents lipid overload in mesenteric macrophages during feeding and in the absence of ANGPTL4 protection these macrophages transform into foam cells associated with chronic inflammatory lesions [158,159,160]. This case story underscores the pivotal importance of understanding the normal biology of the protein to be targeted by a given intervention strategy.

### 4.2. ANGPTL3 Inhibits Lipoprotein Lipase and Endothelial Lipase

Human ANGPTL3 is a 62 kDa glycoprotein expressed in the liver [143,161,162]. ANGPTL3 regulates VLDL levels by inhibiting LPL [163] and plasma HDL levels by inhibiting endothelial lipase (EL) [164]. EL hydrolyses HDL-bound phospholipids and promotes HDL clearance [165,166]. The impact of ANGPTL3 was further studied in a mouse model of atherosclerosis (*ApoE*^−/−^ background), where the *Angptl3*^−/−^ resulted in a significant reduction in the atherosclerotic lesions [7]. This combined effect of ANGPTL3 loss-of-function on LPL and EL regulation was underscored by other investigations finding low circulating concentrations of LDL-C, HDL-C and TGs [162,167,168,169]. Furthermore, human subjects with circulating ANGPTL3 concentrations in the lowest tertile had reduced risk of myocardial infarction compared to subjects in the highest tertile [8].

ANGPTL3 is sensitive to cleavage by proprotein convertases between its N-terminal and C-terminal domain [170], which reduces its potency towards LPL inhibition [171]. ANGPTL3 inhibition of LPL sensitizes LPL to cleavage by proprotein convertases [172] as found with ANGPTL4-mediated LPL inhibition (Dijk 2016; Lund Winther 2021). Exactly how ANGPTL3 inhibits LPL and EL still needs to be clarified, but the first α-helix in ANGPTL3 contains an inhibitory LPL motif [173] proximal to a putative heparin binding motif [164,170,172].

Besides inhibiting LPL and EL, ANGPTL3 may have unrelated pro-atherogenic effects [174] such as arterial wall thickening [175], and promoting angiogenesis via integrin αVβ3 [176].

### 4.3. ANGPTL8 Forms Complexes with ANGPTL3 and ANGPTL4

ANGPTL8 is a 22 kDa protein that has very limited inhibitory effect towards LPL on its own [177,178] and overexpression of ANGPTL8 does not decrease TGs levels in *Angptl3*^−/−^ mice as it does in wt mice [179]. The reason for this difference is the obligate requirement of ANGPTL8 to enter a 1:3 complex with ANGPTL3 to become secreted and gain a high potency in LPL inhibition. ANGPTL8 can also forms a 1:1 complex with ANGPTL4 [180]. While ANGPTL3/8 is a potent inhibitor of LPL, ANGPTL4/8 is a very poor inhibitor and the ANGPTL3/8 and ANGPTL4/8 levels are positively correlated with circulating TGs [180]. In the fed state, ANGPTL4 expression is downregulated in WAT and hepatic secretion of the ANGPTL3/ANGPTL8 complex is increased [146,147,181]. This promotes LPL activity in WAT, and ANGPTL3/ANGPTL8 mediated LPL suppression in heart and skeletal muscle. In the fasted state, this scenario is reversed as *ANGPTL8* is downregulated and *ANGPTL4* upregulated, which provides an increased flux of FA into adipose tissue [148,182].

## 5. Dysfunctional LPL and Hypertriglyceridemia

Homozygous or compound heterozygous individuals with loss-of-function mutations in LPL, ApoC-II, ApoA-V, LMF1, and GPIHBP1 develop a rare monogenic disorder known as familial chylomicronaemia syndrome (FCS). The disorder is defined by an excessive accumulation of chylomicrons with clinical symptoms such as eruptive xanthomas and acute pancreatitis that are associated with very high serum TGs concentrations (>2000 mg/dL) [183,184,185]. The more common polygenic variant of HTG is caused by genetic factors in combination with secondary factors such as type 2 diabetes, obesity, renal disease, pregnancy, and alcohol intake and is characterized by increased plasma levels of TRLs and reduced levels of HDL-C [186]. GWAS identified an association between 32 common gene variants and elevated plasma TGs concentrations and in many cases increased risk of CVD. These variants include ApoA-V, LPL, and ApoB, which are all strong determinants of polygenic HTG [187]. Of the more than 100 *LPL* variants that have been identified, a subset has been classified as pathogenic. The more common *LPL* variants (*LPL-N291A, LPL-D9N,* and *LPL-G188E*) are associated with mildly increased TGs levels [188] and generate an atherogenic lipoprotein profile [5]. On the contrary, another common *LPL* variant with gain-of-function characteristics (*LPL-S447X*), is associated with decreased plasma TGs levels compared to non-carriers [188] and lower risk of CHD [189]. The beneficial properties of this variant were exploited in a LPL gene therapy strategy (Alipogene tiparvovec) for treatment of patients with LPL deficiency. The trials demonstrated transiently reduced fasting plasma triglyceride levels compared to baseline after 12 weeks of administration, and reduction in pancreatitis incidences [190]. Despite these promising results, Alipogene tiparvovec was discontinued as therapy due to excessive costs. Currently, first line of treatment for HTG is dietary and lifestyle interventions and control of secondary factors. Recent developments have nonetheless provided new treatment options for patients with monogenic HTG as exemplified by the approval of Volanesorsen, an ApoC-III targeting drug, for the treatment of adults with FCS [141].

## 6. Clearance of Processed Lipoproteins and Risk of Atherosclerosis

After intravascular processing of the marginated TRLs, the remaining lipoprotein (remnant particle) detaches from the endothelial cells along with some inactive LPL molecules still attached. That inactive LPL acts as a ligand for a number of internalization receptors present on hepatocytes, facilitating the hepatic uptake of remnant particles [191,192]. These receptors include LDL receptor, VLDL receptor, LRP1 and SCD1 [193,194,195,196,197,198,199]. A possible downside of this mechanism, is that, LPL may act to promote LDL and VLDL retention in the subendothelial cell matrix thus delaying their return to the circulation by endothelial transcytosis [200]. Such LPL-mediated LDL retention represents a possible pathogenic factor for the progression of the atherosclerotic lesions as it promotes lipoprotein uptake in macrophages increasing the risk of foam cell formation and inflammation [12]. In the arterial intima, LPL facilitates this temporal sequestering of lipoproteins by its strong ionic interactions with proteoglycans such as biglycan, versican, and collagen XVIII [201,202]. Excessive accumulation of lipoproteins in the subendothelial space leads to fatty streaks, which is a hallmark of early precursor lesions in atherogenesis leading to plaque formation and progression [203,204]. When macrophages are recruited to lipid-laden precursor lesions in the arterial intima, they start phagocytosing the dense lipid depositions and transform into foam cells. This elicits a number of pathogenic responses ultimately leading to chronic inflammation: *(i)* excessive lipid overload in macrophages impairs cholesterol efflux [205] and *(ii)* secretion of pro-inflammatory cytokines and ROS upon macrophage activation by oxLDL [206,207]. Furthermore, these foam cells secrete a number of proteases that degrade the extracellular matrix weakening the plaque structure provoking its rupture with severe cardiovascular damage and risk of thromboembolism [208,209]. Studies in mice show that macrophages display increased LPL expression in early atherosclerosis [210] and the enhanced LPL expression accelerates foam cell formation [211,212]. Importantly, mice with *Lpl*^−/−^ confined to macrophages have reduced lesion sizes and lower diet-induced atherosclerosis [213,214].

## 7. Remnant Particles and Their Role in Atherosclerosis

Very severe HTG characterized by the accumulation of chylomicrons and large VLDLs is generally not considered pro-atherogenic since these particles are too large to be trancytosed across endothelial cells and enter the arterial intima. Remnant lipoproteins, however, with a diameter <70 nm can be transcytosed [215]. IDL and small VLDL particles readily enter the arterial intima and are hence considered atherogenic [129]. The high atherogenic impact of VLDLs and IDLs in arterial intima is underscored by the finding that the majority of ApoB isolated from human atherosclerotic plaques is derived from VLDL and IDL lipoproteins rather than LDL [216]. Studies on hyperlipidemic rabbits show that both VLDL and IDL particles enter and are retained in the arterial intima to the same extent as LDL particles [217]. Other studies claim remnant particles to be more atherogenic than LDL itself [218]. This proposition is underscored by two observations. First, remnant particles do not need to be oxidized before they are taken up by macrophages in the arterial intima [218,219]. Second, they carry approximately 40 times more cholesterol per remnant particle compared to LDL particles [215]. Previously, oxLDL and chylomicron remnants were considered the main atherogenic lipoproteins, but VLDL remnants may have an even greater atherogenic potential [220].

The formation of remnant particles is an integral part of intravascular lipolysis and represents a byproduct of normal lipid homeostasis. Therapeutic interventions preventing the retention of remnant particles in the arterial intima appear more straightforward than limiting the production of remnant particles. One such therapeutic approach is to block or reduce binding of ApoB to HSPGs in the subendothelial spaces. ApoB exists in two isoforms on lipoprotein particles, ApoB-48 and ApoB-100, both of which are encoded by the same gene and play a crucial role in lipid homeostasis [221]. ApoB-100 is a very large protein containing 4536 amino acids [222]. It is expressed in liver and all lipoproteins synthesized by hepatocytes (VLDL) contain ApoB-100 [222]. On the other hand, the shorter isoform ApoB-48 is found on lipoproteins of intestinal origin [223]. ApoB-100 is required for the synthesis, assembly and secretion of TRLs originating from liver. Loss-of-function variants in *APOB* cause hypobetalipoproteinemia, normotriglyceridemic hypobetalipoproteinemia, and hypercholesterolemia—diseases affecting plasma cholesterol and ApoB levels. In aggregate, these observations highlight the potential of ApoB as a clinical biomarker in diagnostics as well as therapeutic target. Mipomersen is an FDA-approved second generation antisense oligonucleotide, that has been designed to reduce plasma concentration of ApoB-100 [224]. Since ApoB-100 is an integrated component of all atherogenic lipoproteins (including LDL), use of Mipomersen may also reduce the risk of atherosclerosis.

### Increase Triglyceride Levels—A Residual Risk in Atherosclerosis

Epidemiological studies suggest that triglycerides and remnant cholesterol are independent risk factors for atherosclerotic cardiovascular disease [225]. Therapeutic interventions have focused on lowering LDL-C levels with statins, cholesterol absorption inhibitors, bile acid sequestrants, and proprotein convertase subtilisin/kexin type-9 (PCSK-9) inhibitors [226]. Statins have proven remarkably efficient in lowering LDL-C [227]. Although, the impressive results from statin therapy are indisputable, they do not represent a universal remedy to patients with atherosclerosis and CVD. Even when treated with the recommended statin levels, some patients require additional lipid lowering therapies and others simply do not respond to statin treatment. One such scenario is patients suffering from familial hypercholesterolaemia, a common genetic disorder, where statin monotherapy does not reach target LDL-C levels [228]. In some cases, even after lowering LDL-C levels to desired level, residual cardiovascular risk still remains, leading to ASCVD [229]. This has led researchers to target atherosclerosis from other therapeutic angles by looking further into disease mechanism(s).

A prime focus has been on the role of HDL-C in atherosclerosis and as a potential therapeutic target for treatment of ASCVD. Low HDL-C has been shown to be a strong independent predictor of premature atherosclerosis, but very high levels of HDL-C were not athero-protective [230]. The role of HDL-C in atherosclerosis is still under debate and it has been suggested that the proportion of dysfunctional HDL versus functional HDL rather than total HDL-C levels may be of importance [231].

Currently, the aim of reducing plasma TGs levels gains momentum as it may have additional benefits in slowing atherosclerotic progression. The association between impaired LPL activity and risk of ASCVD were evaluated in a meta-analysis showing that increased risk of CHD was found in carriers of loss-of-function *LPL* variants [5]. The association between impaired intravascular lipolysis and atherosclerosis was further strengthened by meta-analysis showing that increased levels of plasma triglycerides (TGs) is a risk factor for cardiovascular disease [4] and for coronary artery disease [6]. Residual HTG persist in 20% of U.S. adults with diabetes, including those on statin therapy with well-controlled LDL-C and these patients are at moderate to high 10-year risk for ASCVD [232]. Elevated triglyceride levels are concordantly considered a residual risk factor for the atherosclerosis [225].

## 8. Conclusions

Several different strategies are being exploited for the treatment of severe HTG to reduce the risk of atherosclerosis. This includes monoclonal antibody therapy, antisense oligonucleotide therapy, and gene therapy. The antisense oligonucleotide therapy Mipomersen targeting APOB was approved by FDA in 2013 as lipid-lowering medication. Unfortunately, adverse effects combined with high withdrawal rate due to intolerance led to the withdrawal of Mipomersen in 2019. More promising are the APOC-III targeting antisense oligonucleotide therapies, such as Volanesorsen, which received approval in EU for the treatment of adult patients with FCS [141] and has paved the way for a next-generation of ligand-based antisense oligonucleotide targeting APOC-III (AKCEA-APOCIII-L_RX_). Recently, targeting ANGPTL3 and ANGPTL8 to lower circulating TGs levels in the treatment of dyslipidemia is also pursued. An explorative novel approach is to target dyslipidemia using a variation of the CRISPR-Cas9 platform permanently downregulating *Angptl3* by introducing non-sense mutations [233]. More mature drug development programs targets ANGPTL3 utilizing monoclonal antibody (Evinacumab) or antisense oligonucleotide treatment (ANGPTL3-L_Rx_) and are in clinical phase 2. These drugs retard progression of atherosclerosis in mice and reduce levels of atherogenic lipoproteins in mice, monkeys, and humans [11,234,235,236]. Conclusive data of phase 3 clinical trials are still needed to define their efficacy profiles. Targeting ANGPTL8 is also exploited in early pre-clinical studies with antibodies that neutralize ANGPTL8 and increase cardiac LPL activity and decrease plasma TGs levels in mice and monkeys with beneficial effects on body weight [237,238].

Targeting ANGPTL3 or ANGPTL8 represents promising strategies for the development of new treatment of dyslipidemia. This line of work was recently encouraged by data from combination therapies using statins, antibodies targeting PCSK9, and ANGPTL3 to treat mice with atherosclerotic cardiovascular disease. Statins and PCSK9 antibodies reduce LDL-C plasma levels, while the monoclonal antibody against ANGPTL3 increase VLDL and remnant particle hydrolysis by LPL thereby decreasing the circulating TGs levels in plasma. As only the triple-targeting regime regressed atherosclerotic lesion area and improved lesion composition, this approach seems promising for treating atherosclerosis [239]. These novel approaches may be a future treatment strategy for dyslipidemia when further developed.

## Abbreviations

ANGPTLs	Angiopoietin-like proteins
Apo	Apolipoproteins
ASCVD	Atherosclerotic cardiovascular disease
CCD	Coiled-coil domain
CETP	Cholesteryl ester transfer protein
CHD	Coronary heart disease
CVD	Cardiovascular disease
DSF	Differential scanning fluorimetry
EL	Endothelial lipase
FCS	Familial chylomicronemia syndrome
FFA	Free fatty acids
FLD	Fibrinogen-like domain
GPIHBP1	Glycosylphosphatidylinositol anchored high density lipoprotein binding protein 1
GWAS	Genome-wide association studies
HDL	High-density lipoproteins
HDL-C	High-density lipoproteins cholesterol
HDX-MS	Hydrogen-deuterium exchange coupled to mass spectrometry
HSPGs	Heparans sulfate proteoglycans
HTG	Hypertriglyceridemia
IDL	Intermediate-density lipoprotein
LCAT	Lecithin-cholesterol acyltransferase
LDL	Low density lipoprotein
LDL-C	Low density lipoprotein cholesterol
LDL-R	LDL-receptor
LMF1	Lipase maturation factor
LPL	Lipoprotein lipase
LRP1	LDL receptor related protein 1
LU	Ly6/uPAR protein domain family
PLAT	Polycystin-1, Lipoxygenase, Alpha-Toxin
SCD1	Syndecan-1
Sel1L	Suppressor of lin-12-like protein 1
SKM	Skeletal muscle
SRB1	Scavenger receptor class B type 1
TG	Triglyceride
TRL	Triglyceride-rich lipoprotein
VLDL	Very low-density lipoproteins
WAT	White adipose tissue
